# Novel TLR 7/8 agonists for improving NK cell mediated antibody-dependent cellular cytotoxicity (ADCC)

**DOI:** 10.1038/s41598-021-83005-6

**Published:** 2021-02-08

**Authors:** Vidhi Khanna, Hyunjoon Kim, Wenqiu Zhang, Peter Larson, Manan Shah, Thomas S. Griffith, David Ferguson, Jayanth Panyam

**Affiliations:** 1 Department of Pharmaceutics, University of Minnesota, Minneapolis, USA; 2 Department of Medicinal Chemistry, University of Minnesota, Minneapolis, USA; 3 Masonic Cancer Center, University of Minnesota, Minneapolis, USA; 4 Department of Urology, University of Minnesota, Minneapolis, USA; 5 Center for Immunology, University of Minnesota, Minneapolis, USA; 6 Microbiology, Immunology, and Cancer Biology Graduate Program, University of Minnesota, Minneapolis, USA; 7 Temple University School of Pharmacy, 3307 North Broad Street, Philadelphia, PA 19140 USA

**Keywords:** Molecular medicine, Immunization

## Abstract

There is a significant interest in designing therapeutic agents that can enhance ADCC and thereby improve clinical responses with approved antibodies. We recently reported the combination of an imidazoquinoline-based TLR7/8 agonist (522) with a monoclonal antibody improved ADCC in vitro and in vivo. In the present study, we tested several new small molecule TLR7/8 agonists that induce significantly higher cytokines compared to both the FDA-approved TLR7 agonist, imiquimod, and 522. We evaluated these agonists in combination with monoclonal antibody therapy, with the main goal of enhancing ADCC. Our studies show these TLR7/8 agonists induce robust pro-inflammatory cytokine secretion and activate NK cells. Specifically, we found the agonists 574 and 558 significantly enhanced NK cell-mediated ADCC in vitro as well as enhanced the anti-cancer efficacy of monoclonal antibodies in two different in vivo mouse models. Additionally, we found the agonists were able to stimulate CD8 T cells, likely indicative of an early adaptive immune response*.*

## Introduction

Despite the complex and meticulous design of our immune system, it is sometimes unable to recognize and/or eradicate malignant cells^1^. Additionally, tumor cells can actively suppress immune responses through downregulation of major histocompatibility complex (MHC) class I^2^, antigen shedding^3^, secretion of immunosuppressive cytokines^4^ and upregulation of immunoregulatory checkpoint proteins such as programmed death ligand 1 (PD-L1)^5^. Antibodies have the ability to recognize tumor antigens and direct various components of the immune system against the tumor. The most well-studied antibody mediated mechanism is antibody-dependent cellular cytotoxicity (ADCC), involving natural killer (NK) cells^6^. Several FDA approved monoclonal antibodies mediate ADCC, including cetuximab^7^, rituximab^8^ and trastuzumab^9^. However, not all patients respond to ADCC-based therapies^7,10,11^. A large percentage of the population express the FF isoform of the FcγRIIIa receptor that has poor IgG Fc affinity, resulting in weak ADCC^12–14^. Compounding this issue is the lack of NK cell infiltration into the tumor and inherently low efficacy observed with ADCC^11,15^. Thus, rational combination therapies that leverage ADCC would improve the outcome of antibody-based therapies.

Several groups have reported the application of Toll-like receptor (TLR) agonists, specific for TLR3^16^, TLR7/8^17–19^ and TLR9^20^, to enhance ADCC. We are especially interested in TLR7/8 agonists, because of their ability to directly activate NK cells^21^ and/or dendritic cells^22,23^, which in turn produce a number of NK cell activating cytokines, specifically IL-12, IL-18 and IL-15^24^. NK cell phenotype, i.e. activated versus naïve, is dictated by the delicate balance between the transiently engaged activating and inhibitory receptors. The cytokines released by dendritic cells, in response to TLR7/8 agonists, engage an additional set of activating receptors on NK cells. This results in an activated phenotype in a higher percentage of NK cells, thereby improving ADCC^25^. In a previous report, we formulated 522 (an imidazoquinoline based TLR7/8 agonist) in pH sensitive nanoparticles to enhance NK cell mediated ADCC^26^. In the present study, we evaluated the efficacy of several second-generation, small molecule imidazoquinoline derivates capable of selective activation of TLR7 and/or TLR8^27^ (listed in Table 1) in improving ADCC. The specificity of these compounds has been confirmed using TLR7 and TLR8 reporter cell assays^27^. Our studies show TLR7/8 agonists can be designed to skew cytokine profiles to greatly enhance NK cell activation and improve ADCC with cetuximab (an anti-EGFR monoclonal antibody approved for the treatment of lung cancer)^28^ and an investigational anti-HER2/neu antibody^29^.

**Table 1:** List of synthetic TLR7 and/or TLR8 specific agonists with their EC50 values^27^.

Compound	TLR 7 (μM)	TLR 8 (μM)
522	2.22	9.88
561	3.21	NA
563	2.89	NA
571	NA	49.8
574	0.6	2.21
558	0.18	5.34
543	4.43	14.48

## Results

### TLR7/8 agonists result in improved cytokine induction in human PBMCs

**Figure 1:**
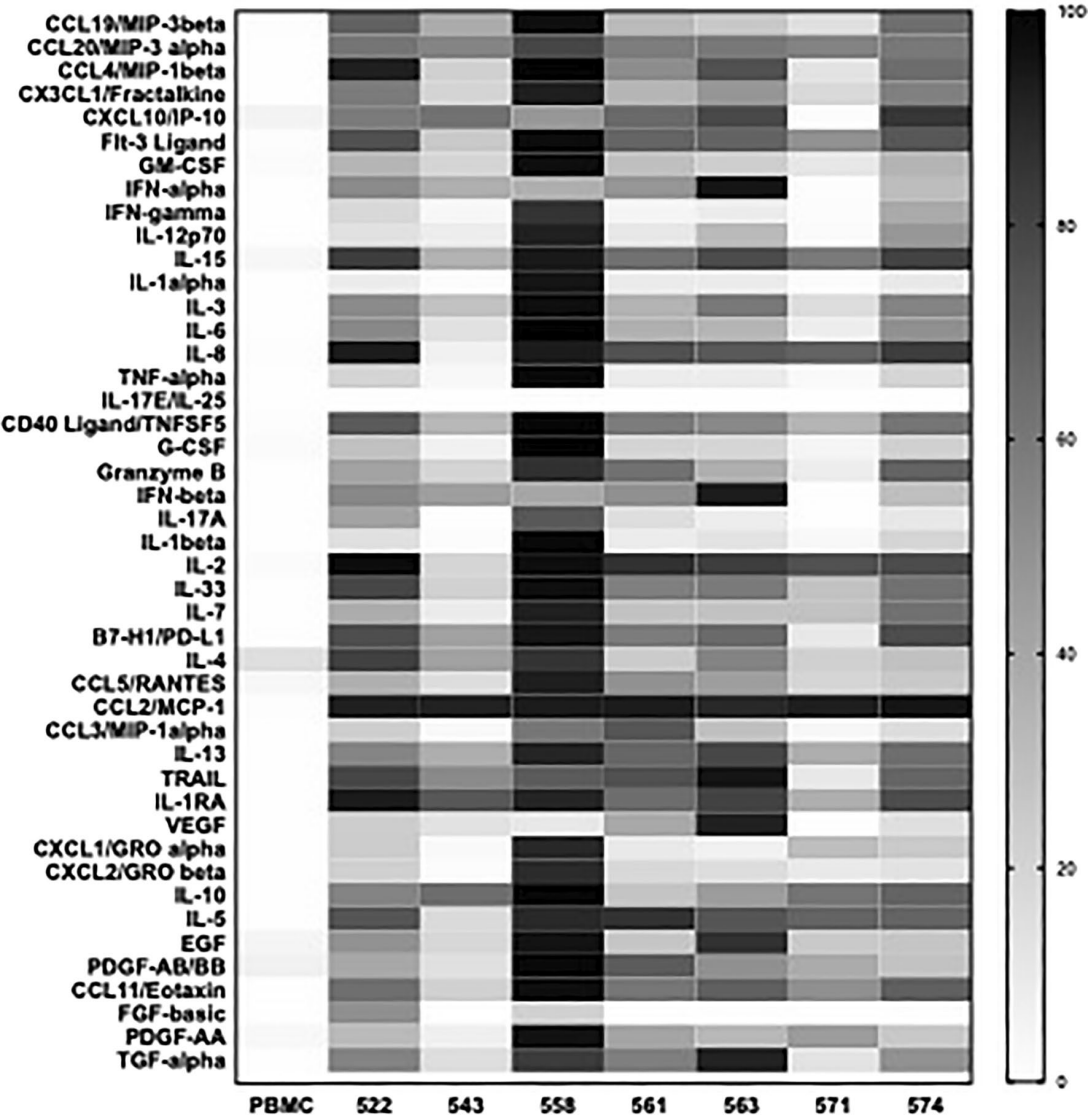
Cytokines secreted by PBMCs (Donor I) upon treatment with the different TLR7/8 agonist compounds (1 μM). Data is scaled from 0 to 100 for each cytokine (0 was assigned to the lowest value and 100 was assigned to the highest value).

One of the key parameters of TLR7/8-mediated activation is the release of inflammatory cytokines by immune cells. Considering both direct and indirect pathways of activation have been reported for TLR7/8 mediated NK cell activation^17,22,23^, we decided to screen our panel of second generation TLR7/8 agonists for their ability to induce cytokine production by human peripheral blood mononuclear cells (PBMCs) in vitro. We tested PBMCs from two healthy donors (Fig. 1 and Supplementary Fig. S1). Several of the compounds, 558 in particular, induced robust cytokine production. Cytokines traditionally thought to be critical for activation of NK cells include IFN-α, IFN-β, IL-2, IL-12 and IL-15^24^. We observed treatment with 558 resulted in significantly higher levels of these cytokines compared to those in the untreated group. Similarly, IFN-γ, a key Th1 cytokine that stimulates T cell activation and is critical for anti-tumor immune responses, was significantly upregulated. IL-10, a cytokine considered to inhibit T cell activation, was also upregulated.

### TLR7/8 agonists increase BMDC activation in vitro

The primary cell type expressing TLR7/8 are dendritic cells (DCs). To evaluate the effect of our second generation TLR7/8 agonists on DCs, we treated mouse BMDCs with the agonists and analyzed the surface expression of the co-stimulatory molecules CD40, CD70 and CD86 using flow cytometry (Supplementary Fig. S2). Due to the limited number of DCs obtained from human blood, we utilized mouse BMDCs to examine co-stimulatory molecules as a proof of concept. In the case of CD70, 558 treatment resulted in significantly higher percentage of activated CD11^+^ DCs compared to any of the other compounds tested. Similarly, 558 treatment resulted in the highest percentage of CD11^+^ DCs expressing CD86. 522, 558, 561 and 563 stimulation, however, resulted in similar levels of CD40 expression, which was higher than that with the other compounds tested.

### TLR7/8 agonists improve cetuximab mediated NK cell degranulation

**Figure 2:**
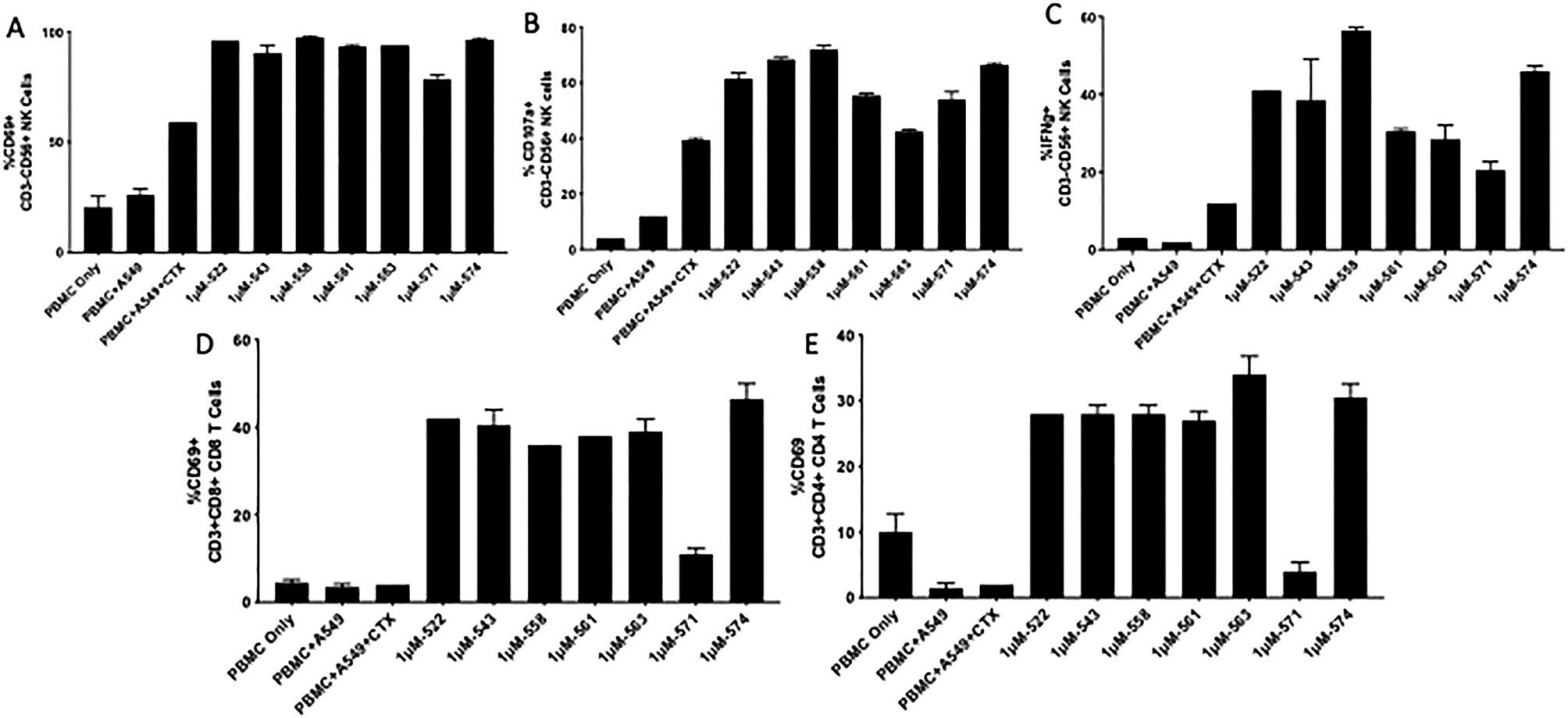
Activation of immune cell subsets in human PBMCs (Donor I) in response to TLR7/8 agonist stimulation. CD3-/CD56 + cells were gated as NK cells. Percentage of NK cells positive for the different markers are plotted on bar graphs. NK cells were then gated for **(A)** the activation marker CD69; all compounds showed significant improvement in CD69 expression when compared with antibody treatment (P < 0.0001). **(B)** the degranulation marker CD107a; all compounds other than 563 showed significant improvement in CD107a expression when compared with antibody treatment (P < 0.0001) and **(C**) the cytokine IFN-γ; all compounds other than 571 showed significant improvement in IFN-γ expression when compared with antibody treatment (P < 0.0001). CD8 and CD4 T cells were gated as CD3^+^/CD8^+^ and CD3^+^/CD4^+^, respectively. **(D)** CD8 T cells were then gated for the activation marker CD69; all compounds except 571 showed significant improvement in CD69 expression when compared with antibody treatment (P < 0.0001). **(E)** CD4 T cells were then gated for the activation marker CD69; all compounds other than 571 showed significant improvement in CD69 expression when compared with antibody treatment (P < 0.0001). All samples other than ‘PBMC Only’ contained A549 (target) cells. All samples other than ‘PBMC Only’ and ‘PBMC + A549’ contained cetuximab (200 nM). Statistical significance was measured by two-way ANOVA with multiple comparisons.

As mentioned previously, NK cell activation can be induced by several different cytokines^25^. Upon activation by TLR7/8 agonists, DCs release cytokines, including those that activate NK cells^24^. Activated NK cells release cytotoxic molecules such as perforin and granzyme through degranulation. We evaluated the ability of our TLR7/8 agonists to induce NK cell degranulation in the presence of a therapeutic monoclonal antibody, cetuximab. Using flow cytometry, we examined CD107a (degranulation marker), IFNγ (cytokine indicative of NK and T cell activation), and CD69 (early activation marker for NK cells) in human PBMCs stimulated with the compounds. CD69 was upregulated in almost the entire population of NK cells tested, for all the compounds tested, and in both donors (Fig. 2A and Supplementary Fig. S4A). This was significantly higher than the baseline levels of CD69 observed in PBMCs, as well as the CD69 levels observed when treated with cetuximab alone.

CD107a and IFN-γ expression, on the other hand, varied depending on the compound as well as the donor. Several compounds were significantly better at inducing degranulation and activation of NK cells, as observed by the expression of CD107a and IFN-γ, respectively (Fig. 2B,C and Supplementary Fig. S4B,C). Specifically, compounds 558, 543, and 574 were significantly better than the other compounds tested. It is interesting to note that different donors responded differently to the various compounds tested. In the case of donor I (Fig. 2), most compounds performed quite well, with 558 and 574 standing out in certain assay read-outs. However, for donor II (Supplementary Fig. S4), compounds 543, 558 and 574 showed significantly improved activity.

### TLR 7/8 agonists increase T cell activation in vitro

The lymphocyte fraction of PBMCs consists primarily of T cells, B cells and NK cells. T cells are key players of the adaptive immune system. We analyzed the expression of CD69, an early activation marker, and IFN-γ, a cytokine secreted by activated T cells, on CD8 T cells (CD3^+^/CD8^+^) and CD4 T cells (CD3^+^/CD4^+^). There was an increase in percentage of T cells activated (CD69^+^) with the addition of TLR7/8 compounds, compared to cetuximab alone (Fig. 2D,E). This was true for both CD8 and CD4 T cells and almost all of the compounds tested (except 571). IFN-γ secretion was observed in less than 5% of the CD8 and CD4 T cells in both donors (Supplementary Figs. S3 and S5).

### Compounds 543, 558 and 574 improve cetuximab-mediated ADCC in vitro

ADCC involves binding of an antibody to its target on a tumor cell and to an NK cell simultaneously, leading to the activation of the NK cell and consequent killing of the tumor cell^30^. Cytokines potentiate this mechanism by increasing the number of NK cells involved in ADCC^30^. We tested compounds that showed significant improvement in the degranulation assay i.e., 522, 543, 558 and 574. We tested the compounds at two effector cell to target cell (E:T) ratios of 10:1 and 20:1. All of them except 522 showed significantly better cytotoxicity (and correspondingly reduced cell survival, Supplementary Fig. S6) as compared to that with cetuximab alone (P < 0.0001 for cetuximab + 522 v/s cetuximab + 558/543/574 at effector to target ratio 20:1, Fig. 3). This aligns well with what was observed in our previous publication where 522 induced ADCC but at significantly higher concentrations (30 μM) than was utilized for these experiments.

**Figure 3:**
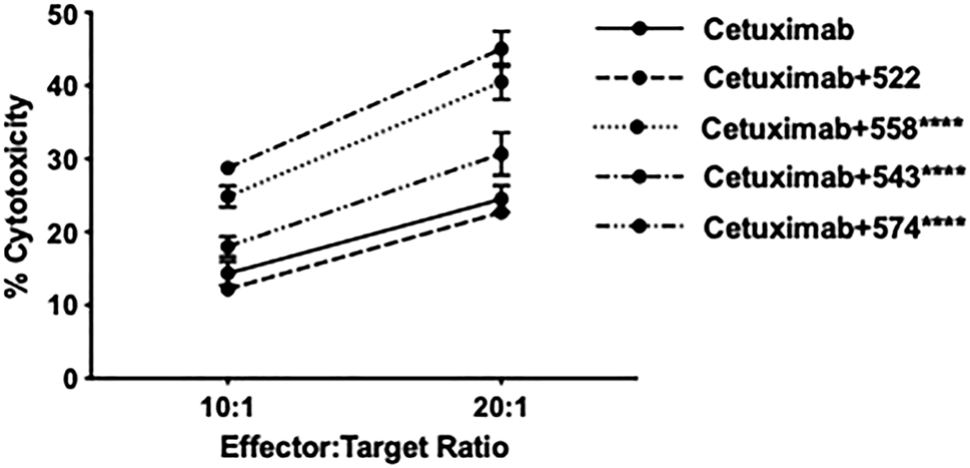
ADCC assay with human PBMCs (LDH based readout). 558, 543, 574 (1 μM) improved cetuximab-mediated ADCC. Percentage cytotoxicity depicted in figure. (****P < 0.0001 for cetuximab v/s cetuximab + 558/543/574 at Effector:Target ratio 20:1, P < 0.001 for cetuximab v/s cetuximab + 558/543 at Effector:Target ratio 10:1, P < 0.05 for cetuximab v/s cetuximab + 574; two-way ANOVA with multiple comparisons).

### Compound 558 enhances tumor growth inhibition in combination with cetuximab in an immunocompromised mouse model

**Figure 4:**
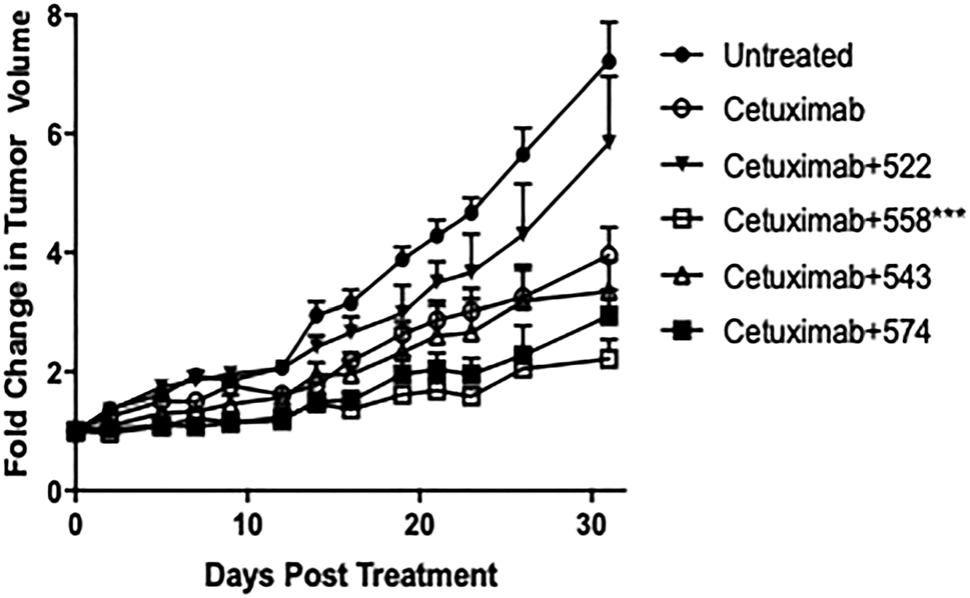
Efficacy study in Balb/c nude mice bearing A549 subcutaneous tumors. Cetuximab + 558 showed significantly improved tumor growth inhibition over Cetuximab alone (P < 0.001 for Cetuximab + 558 v/s Cetuximab alone, two-way ANOVA with multiple comparisons). Statistical significance is based on comparisons from the last day of the study.

To test the in vivo activity of these compounds, we grafted A549 tumors in nude mice and tested them in combination with cetuximab. In alignment with the in vitro ADCC data, compound 522 did not improve the efficacy of cetuximab (Fig. 4). Compounds 574, 558 and 543, on the other hand, showed improved tumor growth inhibition relative to 522, when combined with cetuximab. However, the only compound that outperformed cetuximab alone was 558 (cetuximab + 558 v/s cetuximab alone). 543 was only marginally better than cetuximab alone (not statistically significant). This is particularly interesting because 543 demonstrated the most promising in vitro ADCC activity, suggesting that factors other than NK cell activation alone may play a role in mediating the activity of these agonists. These factors could include differences in the in vivo pharmacokinetics of the compounds.

### Compounds 543, 558 and 574 induce effective antitumor activity in a TuBo Balb/c wild type mouse model

Our in vitro studies using human PBMCs suggested that in addition to NK cell degranulation and ADCC, TLR7/8 agonists also activated T cells. Activation of T cells can lead to memory response and facilitate long-term immunity. To test for the role of T cells, we utilized an orthotopic tumor model where TuBo mammary tumor cells were grafted in wild type Balb/c mice. TuBo cells expresses the HER2/neu antigen and are syngeneic to Balb/c mice^31^. The anti-HER2/neu (termed as anti-neu) monoclonal antibody has been shown to be efficacious in the TuBo model in published studies^29^.

**Figure 5:**
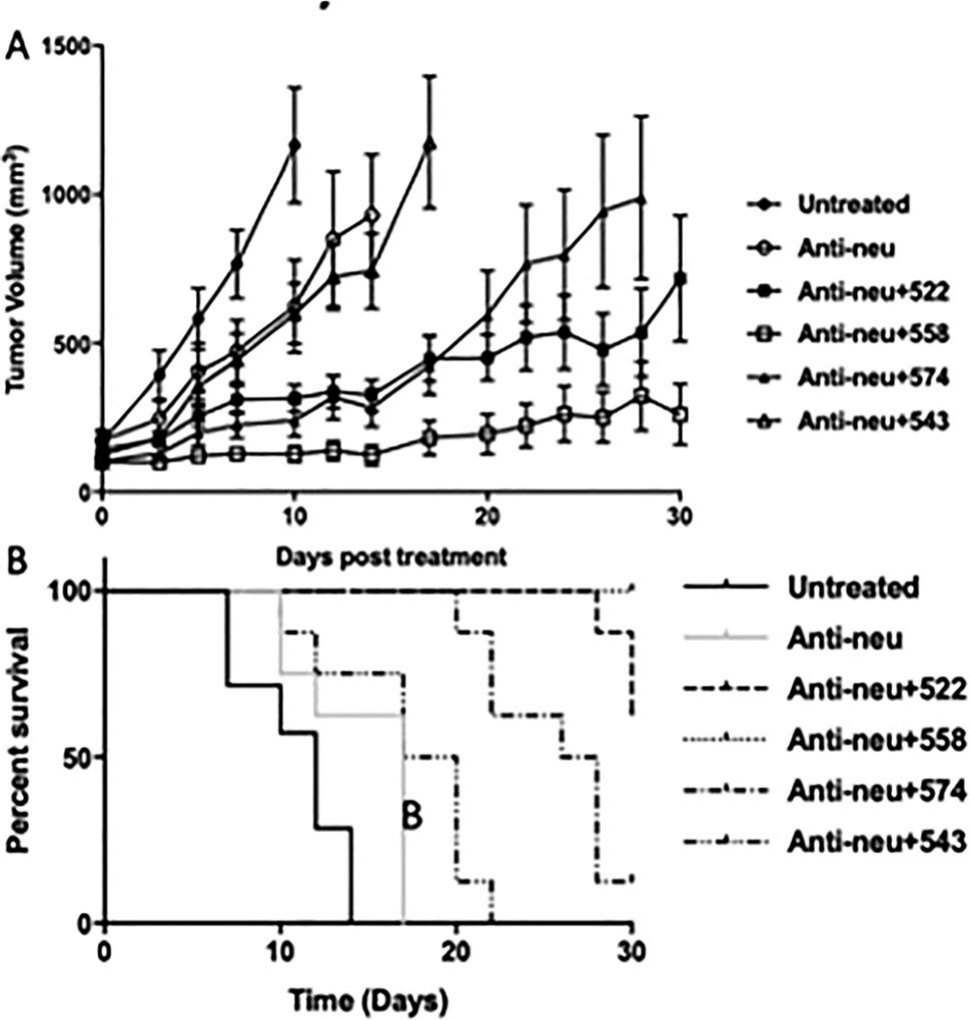
Efficacy study in wild-type Balb/c mice. **(A)** Tumor volumes plotted against time. Administration of the anti-/neu antibody showed significantly improved tumor growth inhibition in comparison to the untreated group. P < 0.0001 for Untreated v/s anti-neu on Day 10. Compounds 522, 558 and 574 performed significantly better in combination with the anti-neu antibody as opposed to the anti-neu antibody alone. P < 0.0001 for anti-neu v/s anti-neu + 522/558/574 on Day 14. P > 0.05 for anti-neu v/s anti-neu + 543 on Day 14. P < 0.01 for anti-neu v/s anti-neu + 558 on Day 30. All statistics were calculated using two-way ANOVA with multiple comparisons. **(B)** Survival curves of mice from the efficacy study. Data for the untreated control and anti-neu antibody treatment group reprinted (adapted) with permission from^26^. Copyright 2021 American Chemical Society.

The anti-neu antibody was only slightly better at tumor growth inhibition than the untreated group (P < 0.0001 for untreated v/s anti-neu on day 10, Fig. 5A). Compounds 522, 558 and 574 were all significantly better in combination with anti-neu antibody as opposed to anti-neu treatment alone (P < 0.0001 for anti-neu v/s anti-neu + 522/558/574 on day 14). Interestingly, 543 (in combination with anti-neu antibody) did not perform significantly better than the antibody treatment alone, a result similar to that observed in the A549 nude mouse model. Tumor was completely eradicated in 1 out of 8 mice treated with anti-neu antibody + 574, and 2 out of the 7 mice in the anti-neu antibody + 558 group. Survival data from this study is presented in Fig. 5B.

### 522 and 558 increase CD8 T cell infiltration into the tumor

**Figure 6:**
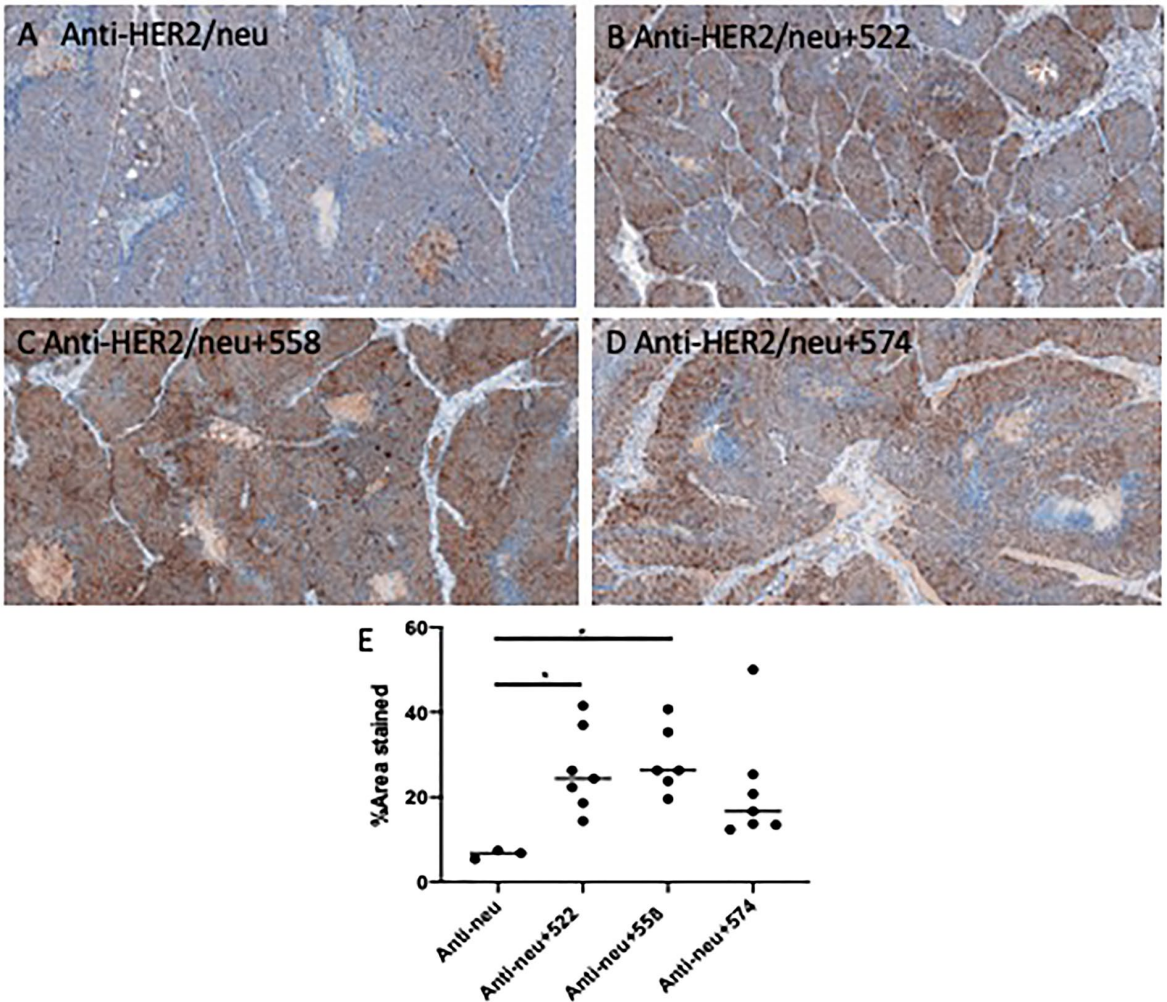
Ex vivo IHC analysis of CD8 T cell infiltration. Representative images presented from each group. Brown is indicative of positive staining. **(A)** Anti-HER2/neu **(B)** Anti-HER2/neu **(C)** Anti-HER2/neu + 558 **(D)** Anti-HER2/neu + 574 **(E)** Quantified data.

In order to understand the immune cell milieu in the tumor, at the end of the study tumors were collected and immune cell infiltration was analyzed through IHC. There was significant T cell infiltration in almost all the sections analyzed (Fig. 6). The addition of 558 or 522 to the anti-neu antibody treatment resulted in significantly higher CD8 T cell infiltration (P < 0.05, Fig. 6B,C,E). It is important to note that in the anti-neu antibody + 558 treatment group, 2 out of 8 mice did not have tumors at the end of the study, and thus we were unable to evaluate T cell infiltration in those animals. The addition of 574 also trended towards an increase in CD8 T cell infiltration; however, the data was not statistically significant.

### Compound 558 increases NK cell activation in the tumor and spleen of wild type Balb/c mice grafted with TuBo tumors

**Figure 7:**
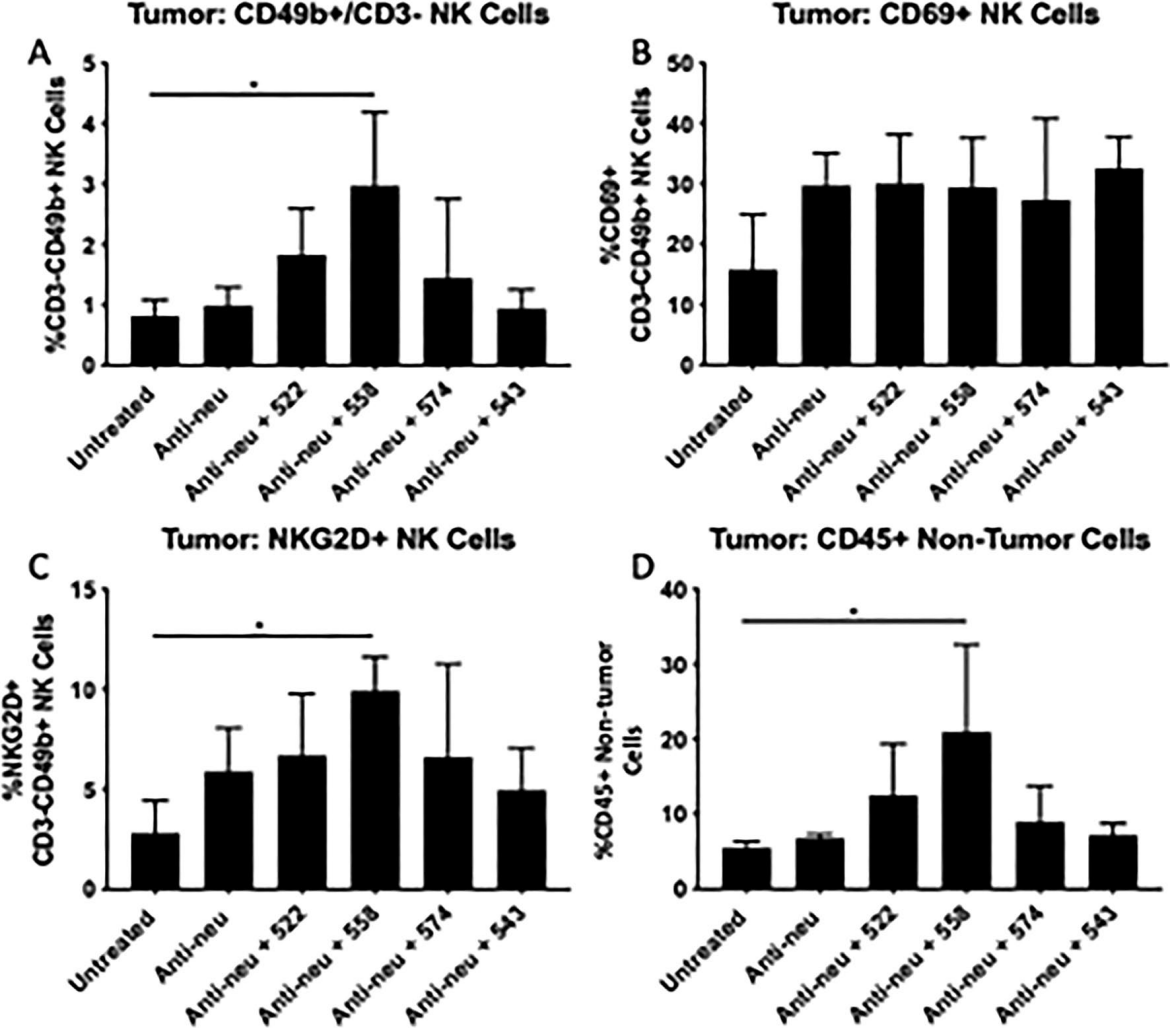
Ex vivo analysis of immune cell infiltration in tumor post treatment with anti-HER2/neu antibody and TLR7/8 agonists **(A)** Percentage of CD49b^+^/CD3^-^ NK cells in tumor, *P < 0.05 **(B)** Percentage of CD69^+^ NK cells in tumor **(C)** Percentage of NKG2D^+^ NK cells in tumor, *P < 0.05 **(D)** Percentage of CD45^+^ ‘non-tumor’ cells in tumor, *P < 0.05. All statistical analysis is based on ordinary one-way ANOVA with multiple comparisons.

To understand the effect of selected agonists on NK cells in vivo at early time points, a separate set of mice were sacrificed three days after the final agonist dose and the immune cell infiltration in the tumor was studied by flow cytometry. Mice that underwent treatment with anti-neu antibody and 558 had a significantly higher percent of NK cells (P < 0.05, Fig. 7A), as well as NKG2D + NK cells (P < 0.05, Fig. 7C) within the tumor—a receptor on NK cells that has been shown to be one of the key triggers in their activation status^25^. The frequency of activated NK cells (gated as CD69^+^ NK cells) in the tumor also increased overall (Fig. 7B), a trend similar to that observed in vitro. Interestingly, the percentage of tumor-infiltrating lymphocytes, as determined by CD45 expression, was also significantly higher in the case of anti-neu antibody + 558 treatment (P < 0.05, Fig. 7D). It is plausible that these CD45 + cells include other immune cells (in addition to NK cells) such as T cells, dendritic cells and macrophages that infiltrated into the tumor and promoted an improved immune response.

In the spleen, the absolute percent of NK cells in the anti-neu antibody + 558 treated group was similar to that observed in the untreated group (Supplementary Fig. S7A). This may be a reflection of a change in the composition of splenocytes in terms of other immune cells, such as T cells. However, the frequency of CD69^+^ NK cells was higher in case of anti-neu antibody + 558 group as compared to that observed in untreated animals (P < 0.05, Supplementary Fig. S7B). There was no change in the percent NKG2D^+^ NK cells in the spleen (Supplementary Fig. S7C).

## Discussion

Monoclonal antibody therapy has improved treatment outcomes in several different cancers. Yet, many limitations exist. A number of different combination therapies are being explored that can further improve the therapeutic efficacy of antibodies such as cetuximab^7^, rituximab^8^ and trastuzumab^32^ that work primarily through NK cell mediated ADCC. Previous reports have described the successful use of TLR7 and TLR8 agonists for augmenting ADCC in vitro^18^. Stimulation of TLRs on dendritic cells leads to an increase in cytokine secretion. These cytokines prime NK cells leading to improved activation^22,23^. Alternatively, TLR7/8 agonists have also been shown to activate NK cells directly^17^. A recent publication from our lab explored the use of a TLR7/8 agonist (522) for improving ADCC^26^. In this previous report, 522 was encapsulated in gas generating nanoparticles (522GGNPs) to enhance delivery to dendritic cells^33^. The combination of 522GGNPs with cetuximab was able to significantly enhance ADCC in vitro and in vivo^26^. It must be noted however that in vitro*,* significantly higher concentrations 30 μM of 522 (in 522GGNPs) were required as compared to the present study where we utilized 1 μM of the compounds. Additionally, in vivo 522 was tested only as a complete formulation i.e. in 522GGNPs. Thus, the present study builds on our prior data by evaluating a panel of second-generation TLR7/8 agonists of higher potency^27^.

NK cells may be activated through cell–cell contact (with dendritic cells) as well as by cytokine mediated signals^24^. Our initial experiments evaluated the effect of the compounds (in combination with the anti-EGFR antibody, cetuximab) on cytokine secretion. We observed significantly higher cytokine induction in vitro, specifically cytokines considered as key drivers of NK cell activation i.e. IFN-α, IFN-β—that activate NK cells, IL-2, IL-15—that promote NK cell survival, proliferation and activation and IL-12—that has been understood to promote optimal cytokine production by NK cells^24^. Additionally several other pro-inflammatory cytokines that contribute to anti-tumor responses were also upregulated, suggesting a strong anti-tumor response (independent of NK cells) could be expected in vivo. Interestingly, IL-10, an anti-inflammatory cytokine was also upregulated with treatment with most of the compounds. This is not an uncommon observation as anti-inflammatory cytokines are often secreted subsequently after pro-inflammatory cytokines to mediate inflammation^34,35^. A temporal analysis of the two will likely provide more insight.

In order to determine the specific source of these cytokines, we examined the effect of 522 and 558 on cytokine expression in immune cell subsets within PBMCs using flow cytometry. We found DCs expressed IL-6 and TNF-α while T cells were positive for IFN-γ in response to 558 treatment (Supplementary Fig. S8). There was no effect of 522 on any of the cell subtypes and none of the agonists had any effect on NK cells. These results do not correlate directly with those from the Luminex-based assay of secreted cytokines (Fig. 1). A number of factors could contribute to the differences in the two results. The flow cytometry-based assay provides a snapshot of intracellular cytokine concentration at a given time while the Luminex assay describes the cumulative amount of cytokine secreted over a certain time window. In addition, differences in the study protocol (such as the use of Brefeldin A to inhibit protein transport in flow cytometry studies) could impact cytokine levels. Thus, additional studies are needed to conclusively determine the specific source of various cytokines.

Next, we evaluated the effect of the TLR7/8 agonists on mouse bone marrow derived dendritic cells (BMDCs) in order to understand the potential for cell–cell contact based activation of NK cells. We observed elevated co-stimulatory molecule expression (CD40, CD70 and CD86) on mouse BMDCs following treatment with these compounds (Supplementary Fig. S2). Co-stimulatory molecules are not only critical components of the DC-T cell immunological synapse but are also critical for NK cell activation. CD70 on DCs can interact with CD27 on NK cells leading to an activated phenotype^36^. Thus, not only did the agonists induce pro-inflammatory cytokines but also enhanced cell–cell stimulation. Our experiments that followed focused on evaluating the phenotype of NK cells in the presence of TLR7/8 agonists.

We observed significantly improved degranulation of NK cells in the presence of TLR7/8 agonists. However, NK cells degranulation may not necessarily result in target cell lysis^37^. In order to understand the effect of NK cells pretreated with TLR7/8 agonists on tumor cells, we performed ADCC assays with the most promising compounds in the degranulation assay (522, 543, 574 and 558). We observed that all compounds other than 522 significantly enhanced ADCC. This likely is reflective of the concentration at which the compounds were tested and the IC50 values. Both 558 and 574 are between two and tenfold more potent than 522 (TLR7 or TLR8 reporter assay). It is unclear though why 543 showed improved activity considering its lower IC50 values. It is plausible that factors such as protein binding differentiate 543 from 522, however this needs to be confirmed through additional experiments.

Through our in vitro assays, we determined the most promising compounds that resulted in enhanced cytokine release and improved ADCC were 543, 574 and 558. It is interesting to note that all three of these compounds were TLR7/8 dual agonists. It has been noted in the literature that although TLR7 and TLR8 stimulation are able stimulate the secretion of a number of overlapping cytokines, TLR7 agonists skew towards interferon regulated cytokines (such as IFN-α) i.e., a Th2 phenotype, whereas TLR8 agonists skew towards pro-inflammatory cytokines TNF-α and IL-12 i.e., a Th1 phenotype^38^. It has also been shown that the receptors for these cytokines are differentially expressed on different subsets of DCs^27^. The combined stimulation of the two receptors likely leads to the stimulation of both Th1 and Th2 immunity, resulting in improved NK cell (and T cell) activation.

The nude mouse model lacks T cells but retains NK cell activity. Our primary goal was to evaluate these compounds for their ability to enhance NK cell mediated ADCC. Thus, the Balb/c nude mouse model was well-suited for this purpose. For this model, the dosing was evenly spaced over a span of sixteen days. This is because NK cells have been understood to undergo short-term activation in response to stimuli, even though there has been recent some debate over this^39,40^. Thus, each cetuximab dose was proceeded with an agonist dose. We found that all three compounds performed significantly well in vivo. In particular, 558 was quite effective in inhibiting tumor growth. Interestingly, 522 (in combination with cetuximab) did not show improved activity over cetuximab alone. This can likely be attributed to poor pharmacokinetic profile of 522 (all previous studies have evaluated 522 in a nanoparticle formulation that provided controlled release of the drug) or due to the lack of T cells in this model (T cells have prolonged activation, unlike NK cells), or both^24^. It is important to note, however, experiments performed in athymic nude mice versus euthymic wild type mice alone do not conclusively establish the role of NK cells. We previously performed cell depletion studies to demonstrate the role of NK cells in mediating the ADCC enhancement activity of one of our lead molecules (522)^26^. Similar studies with the other lead compounds will provide further clarity on the cell types that they activate.

TLR7/8 agonists have been commonly used as vaccine adjuvants, because these compounds activate DCs to secrete a number of cytokines that activate NK cells. However, another mechanism of action of TLR7/8 agonists as anti-cancer vaccine adjuvants has been their indirect activation of T cells. Although the overarching aim of this work was to understand the influence of TLR7/8 agonists on NK cell-mediated ADCC, our in vitro data suggest TLR7/8 agonists also have a strong (and possibly lasting) impact on T cells, in addition to having a strong impact on NK cells. For T cells to mount a specific anti-cancer immune response, not only must they be activated but they must also be presented with the target antigen^4^. Park et al.^29^ identified a link between therapeutic antibodies and adaptive immunity mediated by T cells. They found that an anti-HER2/neu antibody promoted ADCC as its major mechanism but also enhanced cross-priming of T cells, essentially providing T cells with the antigen ‘in situ’. Of note, the paper also reported a memory immune response at high doses of the antibody, very similar to that observed following anti-cancer vaccination. Such effects have also been reported with radiation therapy^41^. Thus, we extended our studies to include a fully immunocompetent model to evaluate T cell responses to combination therapy consisting of monoclonal antibody and TLR7/8 agonists.

For TuBo tumors grafted in Balb/c mice, the dosing was completed within five days, based on our previous experience with immunocompetent models^26^. Compounds 522, 574 and 558 showed effective tumor growth inhibition in combination with the anti-neu antibody, relative to that with the antibody alone. In a fully immunocompetent model, there are at least three parameters that could determine the treatment outcome: (1) pharmacokinetics of the agonist, (2) NK cell activation, and (3) T cell activation. It is possible that 543, although a potent inducer of ADCC, is not a good compound for T cell activation. Similarly, 522 is plausibly better at T cell activation than it is at NK cell activation and thus we see a difference in the outcome between the two tumor models. These studies also provide support to the hypothesis that tumor cell death induced by NK cells creates a rich source of tumor-associated antigens that can potentially activate and prime T cells^29^. Further experiments involving NK and/or T cell depletion are needed to test these possibilities.

Ex vivo analysis of tumors from the TuBo model showed significant NK cell infiltration, especially NK cells with an activated phenotype. Tumors can downregulate the expression of NKG2D in order to side-step immune recognition^15^. It is promising to note that TLR7/8 agonists are able to increase the expression of this key receptor, potentially paving the way for improved immunosurveillance. We also observed an increase in the activated NK cells in the spleen of mice treated with the agonists. Finally, upon examination of the tumor sections at the end of the study, we saw significantly higher T cell infiltration in the tumors of mice that were treated with 522 and 558, thus suggesting the involvement of the adaptive immune system.

Although Park et al.^29^ were successful at eradicating experimental tumors in the preclinical setting, this is often not replicated in the clinic. Currently, despite the success of monoclonal antibody therapy, some patients relapse^43^. Our rationale behind combining the novel TLR7/8 agonists with a therapeutic antibody in an immunocompetent model was to stimulate an intense adaptive immune response^44^. In our studies, although there were several indications of a strong T cell response with the TLR7/8 agonists, tumors were eradicated in only a small fraction of treated mice. This could be because we investigated a single dose of the antibody, whereas Park et al. used between 2 and 4 doses^29^. Thus, further dose refinement may be needed to realize the full potential of the agonists. Although the data presented here is indicative of an early adaptive response, it remains to be tested whether the simple combination of a monoclonal antibody and a TLR/7/8 agonist can provide lasting anti-cancer efficacy. It is interesting to note that several groups have also reported ‘memory’ NK cells when exposed to haptens, viruses and or an inflammatory milieu^42^. Specifically in case of memory responses in an inflammatory milieu, NK cells with prior exposure to IL-12, IL-15 and IL-18 retain the ability to secrete low levels of IFN-γ for a prolonged duration as well as elevated IFN-γ levels upon restimulation^42^. Considering the proposed mechanism of action for TLR7/8 agonists in this study, it would be interesting to study their effects on memory NK cells in future studies. Additionally, understanding the pharmacokinetic behavior of these compounds would also be helpful in further dose optimization. Studies in humanized mouse models that allows evaluation of these agonists in combination with clinical antibody candidates will provide further insight regarding the translational potential of these novel compounds.

## Conclusion

The goal of the present study was to evaluate second-generation TLR7/8 agonists in combination with monoclonal antibodies to improve ADCC. The TLR7/8 agonists 543, 574 and 558 showed promising in vitro ADCC activity. These molecules were able to effectively retard tumor growth in vivo in combination with cetuximab in a heterotopic nude mouse model and in combination with an anti-neu antibody in a syngeneic mammary tumor model in Balb/c mice. Dual TLR7/8 agonists were more effective than TLR7 or TLR8 specific agonists in stimulating pro-inflammatory cytokine secretion and subsequently in inducing ADCC. Additionally, these compounds improved infiltration of NK cells as well as CD8 T cells into the tumor. In vivo studies in immunocompromised and immunocompetent mouse models showed that compound 558 can significantly improve the anticancer efficacy of therapeutic antibodies. Although our work presented here provides insight into possible mechanism for 558, further studies are required to clearly delineate the mechanism(s) of action for 558 in combination with therapeutic antibodies. These studies should include pharmacokinetic characterization, purified immune cell assays in vitro, immune cell depletion studies in vivo and dose optimization.

## Methods

The study was approved by University of Minnesota and were performed in accordance with the relevant guidelines and regulations. In addition, the study was carried out in compliance with the ARRIVE guidelines.

### Materials

All cell culture supplies were obtained from Invitrogen (ThermoFisher Scientific, Waltham, MA), unless otherwise specified. Fetal bovine serum (FBS) was purchased from Atlanta Biologicals (Flowery Branch, GA). All TLR7/8 compounds were synthesized and characterized as previously reported^27^. Frozen human PBMCs were purchased from Cellular Technology Limited (Shaker Heights, OH). Fluorophore labelled anti-human monoclonal antibodies (CD3-FITC, CD56-PE/Cy7, CD11c-PE/Cy7, CD19-PE, GranzymeB-PE, IL-6-APC, TNF-α-APC/Cy7, IFNγ-BV421 and IL-2-BV605) and Brefeldin A were purchased from Biolegend (San Diego, CA).

### Cell culture

A549 lung cancer cells were purchased from the American Type Culture Collection (ATCC, Manassas, Virginia). TuBo breast cancer cells were kindly provided by Dr. Wei-Zen Wei (Wayne State University). Both tumor cell lines were cultured in RMPI supplemented with 10% FBS and 1% Penicillin/Streptomycin. PBMCs were cultured in RPMI supplemented with 10% fetal bovine serum, 100 μg/mL streptomycin, 100 U/mL penicillin.

### PBMCs cytokine secretion

PBMCs were purified from human blood using Ficoll density gradient medium (GE Healthcare) and seeded at 1.5 million cells per well in a 6-well plate. TLR agonists were added to the wells at 1 μM and incubated overnight at 37 °C. The next day, cells were collected and centrifuged at 1200 RPM for 5 min. All samples were set up as duplicates. The supernatant was collected and frozen at − 80 °C until analyzed using the Luminex Human XL Cytokine Discovery Panel (R&D Systems). The cells were used for analysis of NK cell degranulation and T cell activation. Cytokine analysis was performed by the Cytokine Reference Laboratory (University of Minnesota).

For flow cytometry studies, PBMCs were thawed and seeded at 1 million cells/mL per well in a 6 well-plate. 522 and 558 were added to the wells at 1 μM and incubated for 6 h or 24 h at 37 °C. Cells were incubated for 6 h with Brefeldin A for analysis of intracellular cytokines. After incubation, non-adherent cells were collected and stained for T cells (CD3^+^), NK cells (CD56^+^) or DCs (CD11c^+^/CD19^−^). Once stained, the cells were fixed and permeabilized and the fluorophore conjugated monoclonal antibodies for the intracellular cytokines were added. The cells were stained according to the manufacturer's recommendations and were analyzed by flow cytometry.

### BMDCs in vitro assay

Bone marrow derived dendritic cells (BMDCs) were derived using a method described previously^33^. Mature BMDCs were cultivated with 1 μM of selected TLR agonists for 48 h at 37 °C, stained and then analyzed by flow cytometry for activation markers. For all the flow experiments, dead cells were excluded through appropriate gating strategy.

### In vitro ADCC assay

ADCC assay was performed using a modified version of an assay reported previously^18,19,45^. PBMCs were isolated from human blood (healthy donor) using Ficoll density gradient medium (GE Healthcare) and seeded at 0.5 million/ml in RPMI (10% FBS, 1% Pen/Strep) in 6-well plates. TLR agonists were added to the cells at 1 μM and incubated overnight. Target cells (A549) were pre-labeled with 8 µM CFSE (Biolegend, San Diego, CA) and plated in 96-well plates on the day of PBMC isolation. The next day, cetuximab was added to the wells at 200 nM for 1 h and washed gently. Next, treated PBMCs were counted and added to target cells at the specified ratios and incubated overnight at 37 °C. All samples were set up in triplicates. The supernatant was then analyzed using Pierce LDH Cytotoxicity Assay (Thermofisher, Waltham, MA), and the tumor-cell associated fluorescence intensity was determined at excitation and emission wavelengths of 492 nm and 517 nm, respectively, using Spectramax i3x (Molecular Devices, San Jose, CA).

### NK Cell degranulation and T cell activation

Healthy human PBMCs were seeded at 0.5 million cells/ml in RPMI (10% FBS, 1% Pen/Strep) in a 24-well plate. TLR agonists were incubated with the PBMCs overnight at 1 μM. The supernatant was collected and analyzed for cytokine concentrations. Target cells were trypsinized and added to the wells at an effector to target ratio of 2:1. Cetuximab was added at a final concentration of 200 nM. Anti-CD107a APC Cy7 (Biolegend, San Diego, CA) was added to all samples. The samples were incubated at 37 °C. One hour later, Brefeldin A (Biolegend, San Diego, CA) was added and the samples were once again incubated at 37 °C. Four hours later, cells were collected and stained for extracellular markers using anti-CD3 FITC, anti-CD56 Brilliant Violet 650, anti-CD4 PE-Cy7, anti-CD8 Brilliant Violet 605, and anti-CD69 PerCP-Cy 5.5 (Biolegend, San Diego, CA) antibodies. Intracellular staining was performed with anti-IFN-γ APC antibody (Biolegend, San Diego, CA) using eBioscience Foxp3 Transcription Factor Staining Buffer Set (Thermofisher, Waltham, MA). All samples were set up in duplicates. Samples were analyzed using a BD Fortessa H0081 flow cytometer at the University of Minnesota Flow Cytometry Core Facility.

### In vivo efficacy studies

All experimental protocols using animals were reviewed and approved by the University of Minnesota Institutional Animal Care and Use Committee (IACUC) and were performed in accordance with the relevant guidelines and regulations. In addition, the study was carried out in compliance with the ARRIVE guidelines. Balb/c athymic nude mice (Strain 002019, The Jackson Laboratory) were used to graft heterotopic A549 tumors. A549 cells were counted, re-suspended in saline and diluted 1:1 with Matrigel (Corning, Tewksbury, MA). Two million cells in 100 µL were injected subcutaneously. Tumor volumes were measured three times a week, using a digital Vernier calipers (Marathon Watch, Vaughn, Canada). Tumor volume was calculated using the formula (L × W^2^)/2, L being the longer measurement. Treatments began when tumors reached 100 mm^3^ (n = 5–6 per treatment group). Cetuximab (Merck, Dermstadt, Germany) was dosed at 10 mg/kg every fourth day, starting day 0, for five doses (Days 0, 4, 8, 12, 16) by IV tail vein injections. TLR7/8 agonists were dosed at 2 mg/kg (free drug in DMSO diluted with saline) every fourth day, starting day 1 for five doses (days 1, 5, 9, 13, 17) through IP injections (n = 6–7 mice per group).

Wild type Balb/c mice (Strain 000651, The Jackson Laboratory) were used to graft orthotopic TuBo tumors^29^. TuBo cells were counted, re-suspended in saline and diluted 1:1 with Matrigel (Corning, Tewksbury, MA). Two hundred thousand cells in 100 µL were injected subcutaneously in the fourth mammary fat pad. Tumor volumes were measured three times a week. Treatments began when tumors reached 100 mm^3^ (n = 7–8 per treatment group). Tumor volume was calculated using the formula (L × W^2^)/2, L being the longer measurement. Anti-HER2/neu antibody (Clone 7.16.14, BioXcell, West Lebanon, NH) was dosed at 5 mg/kg, on day 0, through IV tail vein injection. TLR7/8 agonists were dosed at 2 mg/kg (free drug dissolved in DMSO and diluted with saline) every day, for five doses, starting day 1 (days 1, 2, 3, 4, 5) through IP injections (n = 7–8 mice per group). Mice were sacrificed when the group average tumor volume exceeded 1000 mm^3^ or on day 30 of the study, whichever was earlier. We note here that this study was done in conjunction with a previously published study^26^. The untreated control and anti-neu antibody treatment groups were common to both studies. Data for the two groups reproduced here with permission.

### Ex vivo analysis of NK cells

Wild type Balb/c mice were grafted with orthotopic TuBo tumors as described above. Treatment began when tumors reached 100 mm^3^. The dosing scheme was identical to that described above (n = 3–4 mice per group). On day 8, mice were sacrificed, tumor and spleen were excised and collected into HBSS (Genesee Scientific, San Diego, CA). Tissues were processed to obtain single cells using a previously described protocol^46^. Tissues were homogenized using GentleMACS (Miltenyi Biotec, Germany) in MACS C Tubes (Miltenyi Biotec, Germany), as per manufacturer’s protocol. Liberase TH Research Grade (0.026 Wunsch units/mL, Sigma-Aldrich, St. Louis, MO) and DNAase I (0.015 mg/ml, Sigma-Aldrich, St. Louis, MO) were added to the samples and incubated for 15–30 min to allow for dissociation at 37 °C. Enzymes were neutralized using IMDM (Life Technologies, Grand Island, NY), cells were washed once and re-suspended in PBS (0.5% BSA, 2 mM EDTA) and filtered through a 100 µm mesh (Fisher Scientific, Hampton, NH). ACK Lysis Buffer (Life Technologies, Grand Island, NY) was used for RBC lysis. Samples were re-suspended in FACs buffer and 5–10% of the sample was stained with anti-CD3 APC-Cy7, anti-CD49b PE-Cy7, anti-CD8 FITC, anti-CD69 Brilliant Violet 605, anti-NKG2D APC, anti-CD45 PerCP-C5.5 (tumor samples only) antibodies (Biolegend, San Diego, CA) for flow cytometry. Samples were analyzed using a BD Fortessa H0081 flow cytometer at the University of Minnesota Flow Cytometry Core Facility.

### Ex vivo immunohistochemistry (IHC)

Wild type Balb/c mice bearing TuBo tumors were treated as described above. Mice were sacrificed when the average tumor volume for the group exceeded 1000 mm^3^ or on day 30 of the study, whichever came earlier. For CD8 T cells, tumors were excised and formalin fixed for 48 h. The tissues were then stored in 70% ethanol until processed. The samples were submitted to the University of Minnesota Comparative Pathology Shared Resource for IHC processing. The sections were stained for CD8 T cells using an anti-mouse CD8α antibody (Catalog #MA5-17594, Thermofisher Scientific, MA). The slides with stained sections were scanned using a slide scanner. Three snippets of approximately 1700 μm × 800 μm dimensions were selected at random in the slide field, at × 10 magnification and quantified for positive staining using the ImageJ software.

## Supplementary Information

Supplementary Information.
