# Supplementary material for: Novel TLR 7/8 agonists for improving NK cell mediated antibody-dependent cellular cytotoxicity (ADCC)
Source: Sci Rep. 2021 Feb 8;11:3346. doi: 10.1038/s41598-021-83005-6 (PMC7870826; doi:10.1038/s41598-021-83005-6)
Supplement: Supplementary file 1 — Supplementary Information. [file 41598_2021_83005_MOESM1_ESM.docx]

Novel TLR 7/8 agonists for improving NK cell mediated antibody - dependent cellular cytotoxicity (ADCC)

Vidhi Khanna^1^, Hyunjoon Kim^1^, Wenqiu Zhang^1^, Peter Larson^2^, Manan Shah^1^, Thomas S. Griffith^3,4,5,6^, David Ferguson^2^, and Jayanth Panyam^1,7^

^1^Department of Pharmaceutics, ^2^Department of Medicinal Chemistry, ^3^Masonic Cancer Center, ^4^Department of Urology, ^5^Center for Immunology, ^6^Microbiology, Immunology, and Cancer Biology Graduate Program University of Minnesota, Minneapolis USA, ^7^Temple University School of Pharmacy, Philadelphia, Pennsylvania USA

**Supplementary Information**

**
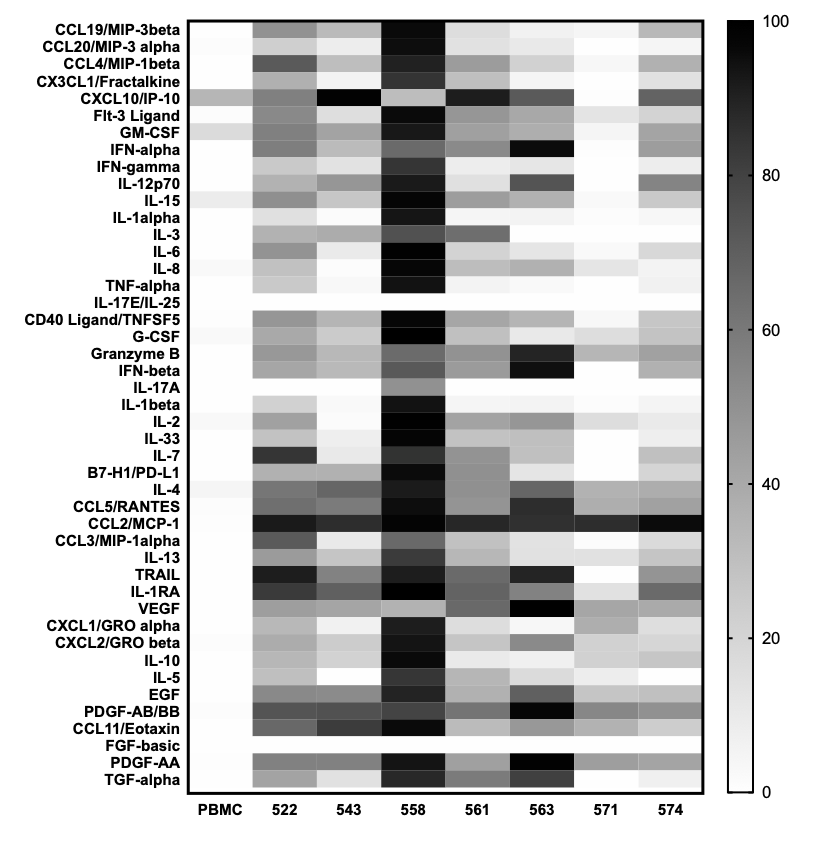
**

**Supplementary Figure 1**: Cytokines secreted by PBMCs (Donor II) upon treatment with the different TLR7/8 agonist compounds (1μM). Data is scaled from 0-100 for each group (0 was assigned to the lowest value in the group and 100 was assigned to the highest value in the group)

**
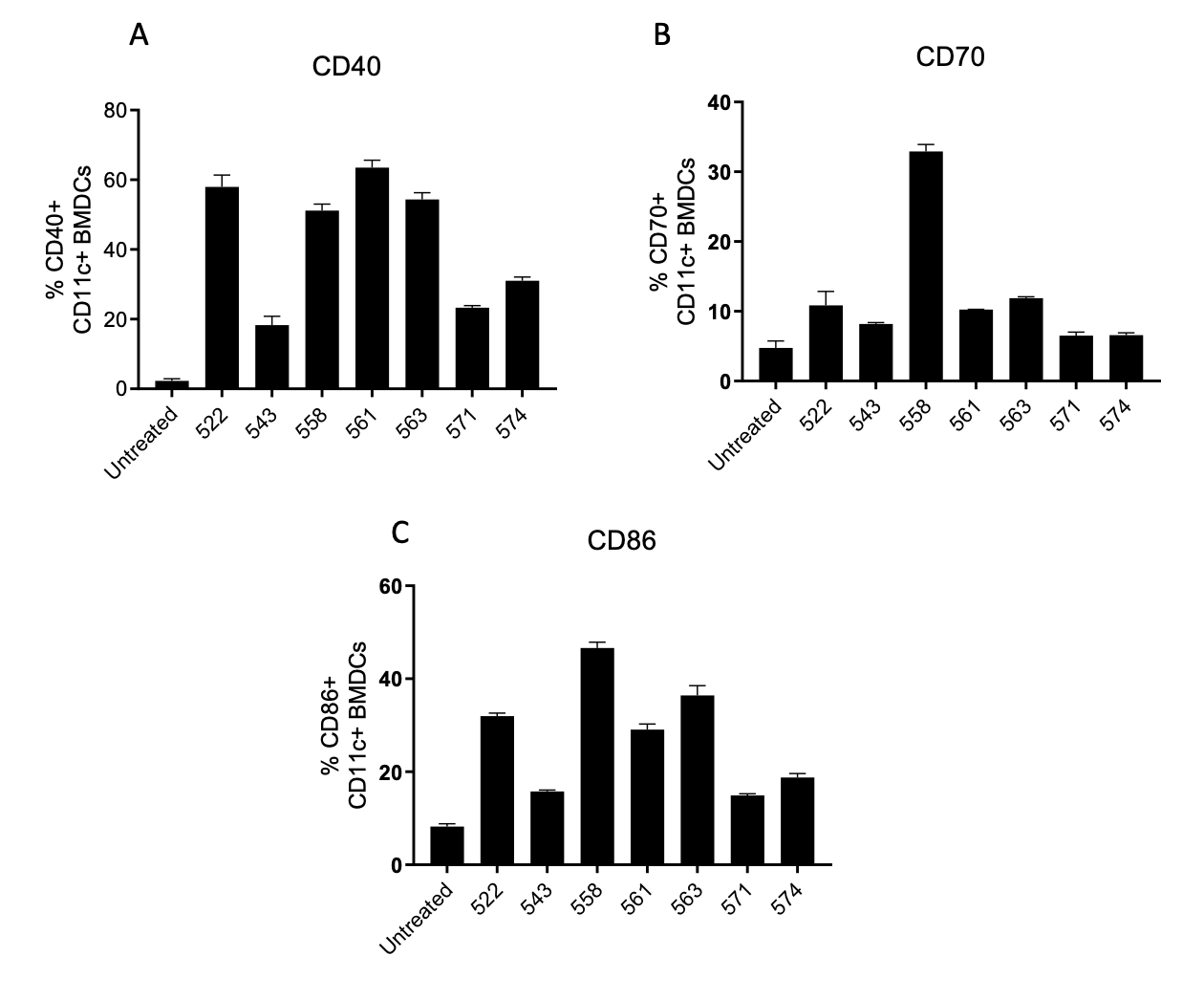
**

**Supplementary Figure 2:** *In vitro* analysis of co-stimulatory markers on mouse bone marrow derived dendritic cells (BMDC). (A) CD40, all compounds significant improvement in CD40 expression (P<0.0001) (B) CD70, all compounds other than 571 and 574 showed significant improvement in CD70 expression (P<0.0001 for 522, 558, 561, 563; p<0.01 for 543) (C) CD86, all compounds significant improvement in CD86 expression (P<0.0001). BMDCs were cultivated with 1 uM of TLR agonists for 48 hrs, then analyzed by flow cytometry. Statistical significance was analyzed using ordinary one-way ANOVA.

**
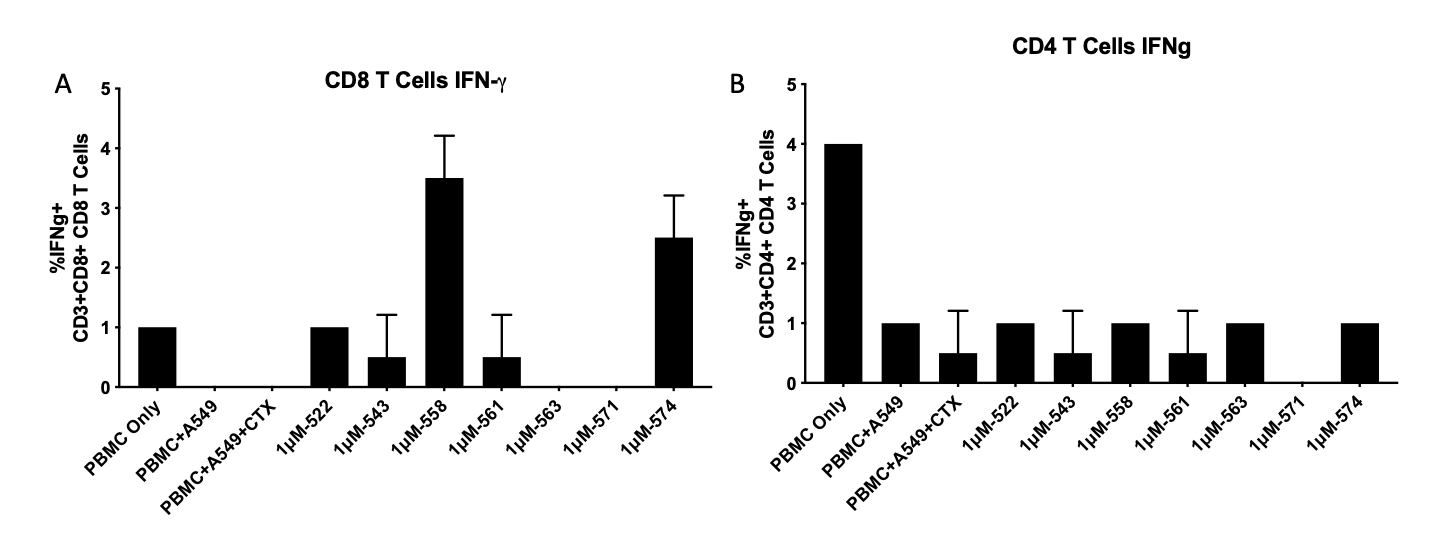
**

**Supplementary Figure 3**: NK cell degranulation assay with human PBMCs (Donor I) using flow cytometry. **(A)** CD8 T cells were gated for the cytokine IFN-γ; no statistical significance was observed. **(E)** CD4 T cells were also gated for the cytokine IFN-γ; no statistical significance was observed. All samples other than ‘PBMC Only’ contained A549 (target) cells. All samples other than ‘PBMC Only’ and ‘PBMC+A549’ contained cetuximab (200nM). Statistical significance was measured by two-way ANOVA with multiple comparisons.

**
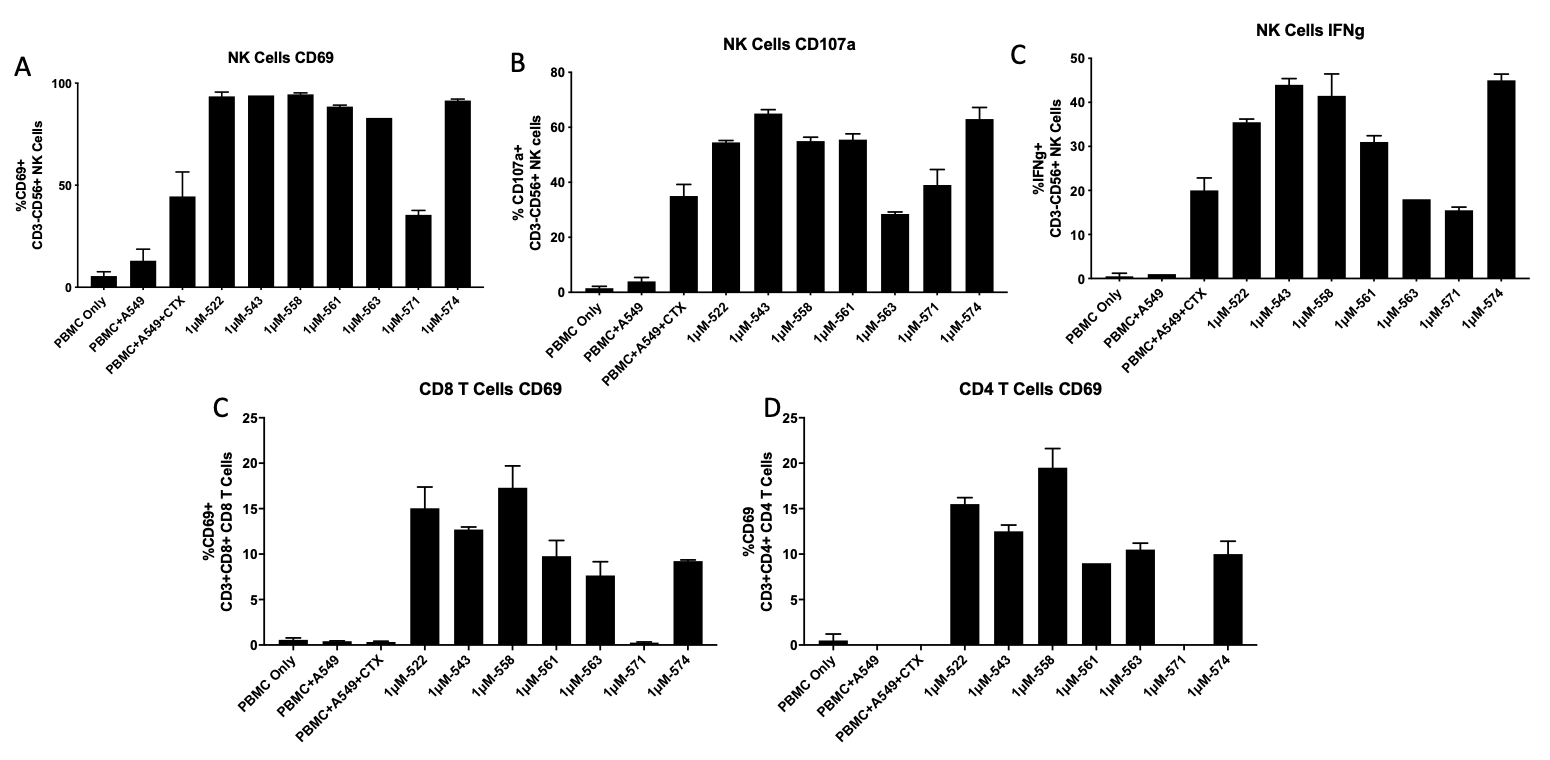
**

**Supplementary Figure 4**: Activation of immune cell subsets in human PBMCs (Donor II) in response to TLR78 agonist stimulation (Donor II) using flow cytometry. CD3-/CD56+ cells were gated as NK cells. Percentage of NK cells positive for the different markers are plotted on bar graphs. NK cells were then gated for **(A)** the activation marker CD69; all compounds showed significant improvement in CD69 expression when compared with antibody treatment (P<0.0001 for all compounds other than 571, P<0.001 for 571). **(B)** the degranulation marker CD107a; all compounds other than 571 showed significant improvement in CD107a expression when compared with antibody treatment (P<0.0001 for all compounds other than 563, P<0.05 for 563) and **(C**) the cytokine IFN-γ; all compounds other than 563 and 571 showed significant improvement in IFN-γ expression when compared with antibody treatment (P<0.0001). CD8 and CD4 T cells were gated as CD3^+/^CD8^+^ and CD3^+^/CD4^+^ respectively **(D)** CD8 T cells were then gated for the activation marker CD69; all compounds other than 571 showed significant improvement in CD69 expression when compared with antibody treatment (P<0.0001 for all compound other than 563, P<0.01 for 563). **(E)** CD4 T cells were then gated for the activation marker CD69; all compounds other than 571 showed significant improvement in CD69 expression when compared with antibody treatment (P<0.0001 for all compounds other than 561, P<0.001 for 561). All samples other than ‘PBMC Only’ contained A549 (target) cells. All samples other than ‘PBMC Only’ and ‘PBMC+A549’ contained cetuximab (200nM). Statistical significance was measured by two-way ANOVA with multiple comparisons.

**
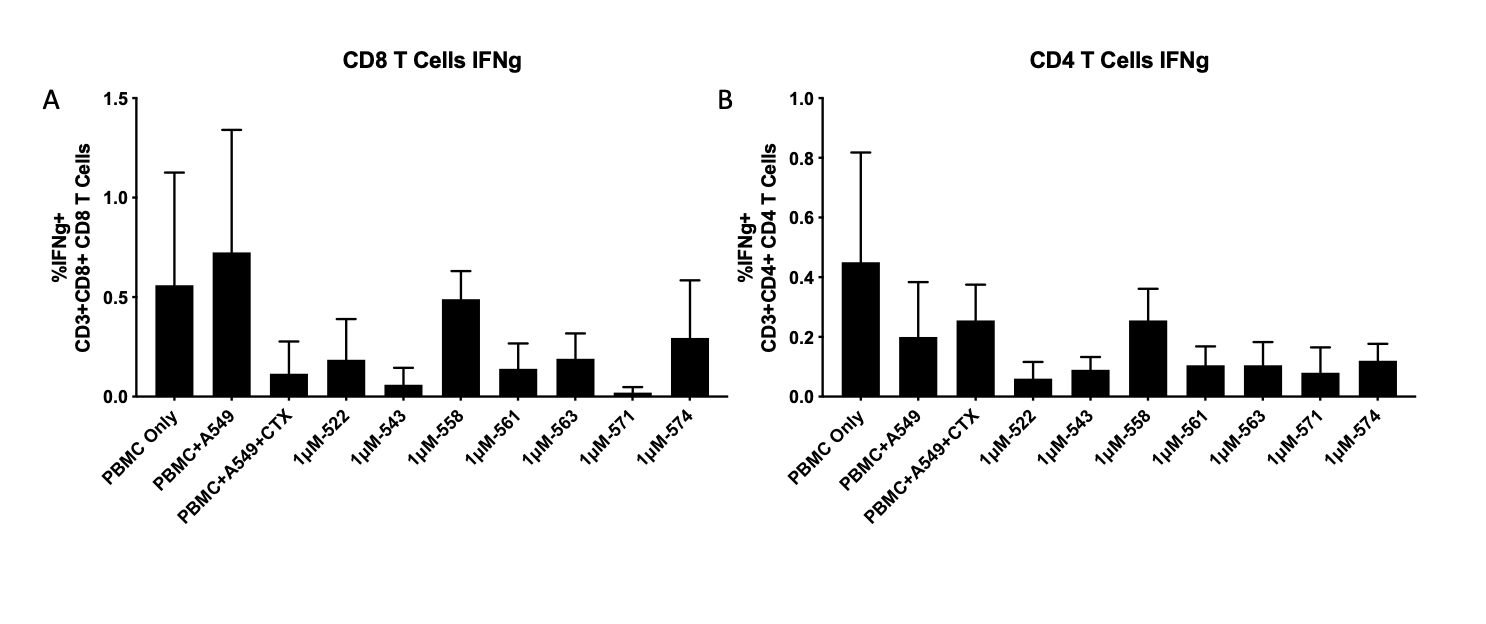
**

**Supplementary Figure 5**: NK cell degranulation assay with human PBMCs (Donor II) using flow cytometry. **(A)** CD8 T cells were gated for the cytokine IFN-γ; no statistical significance was observed. **(E)** CD4 T cells were also gated for the cytokine IFN-γ; no statistical significance was observed. All samples other than ‘PBMC Only’ contained A549 (target) cells. All samples other than ‘PBMC Only’ and ‘PBMC+A549’ contained cetuximab (200nM). Statistical significance was measured by two-way ANOVA with multiple comparisons.

**
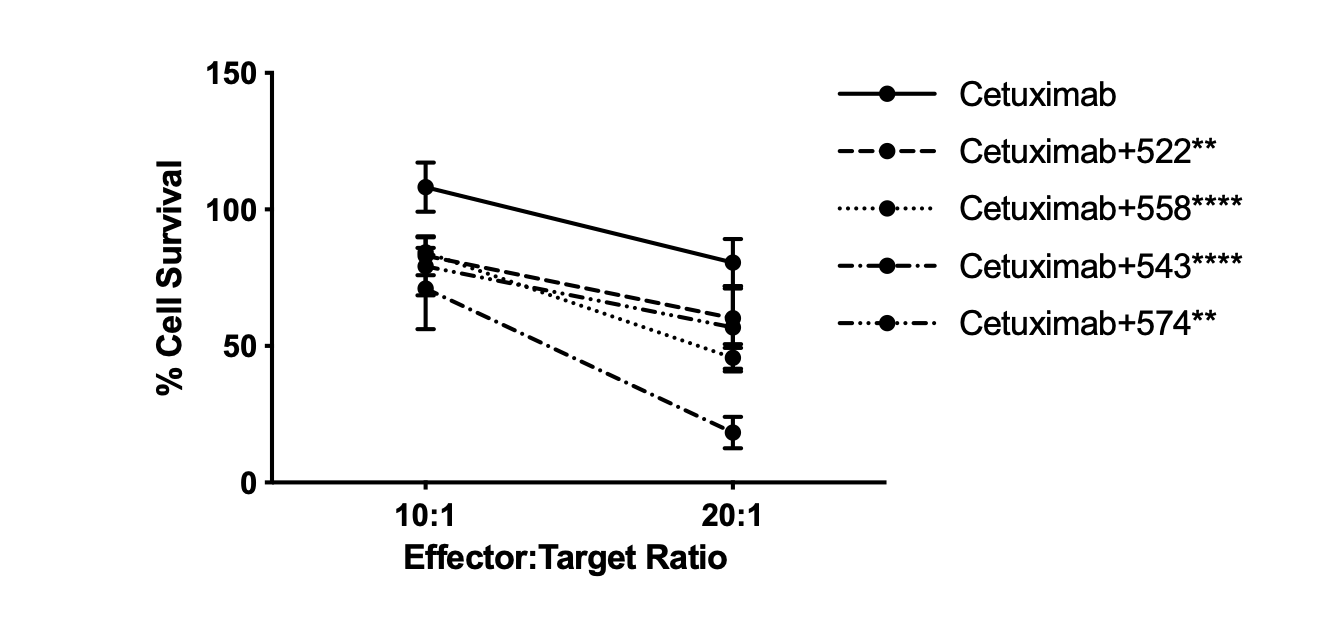
**

**Supplementary Figure 6**: ADCC assay with human PBMCs (CFSE based read-out). 558, 543, 574 (1μM) improved Cetuximab mediated ADCC. Percentage cell survival depicted in figure. (****P<0.0001 for Cetuximab v/s Cetuximab+558/543 at Effector:Target ratio 20:1, P<0.01 for Cetuximab v/s Cetuximab+522/574 at Effector:Target ratio 20:1, statistical significance at Effector:Target ratio 10:1 not shown; two-way ANOVA with multiple comparisons).

**
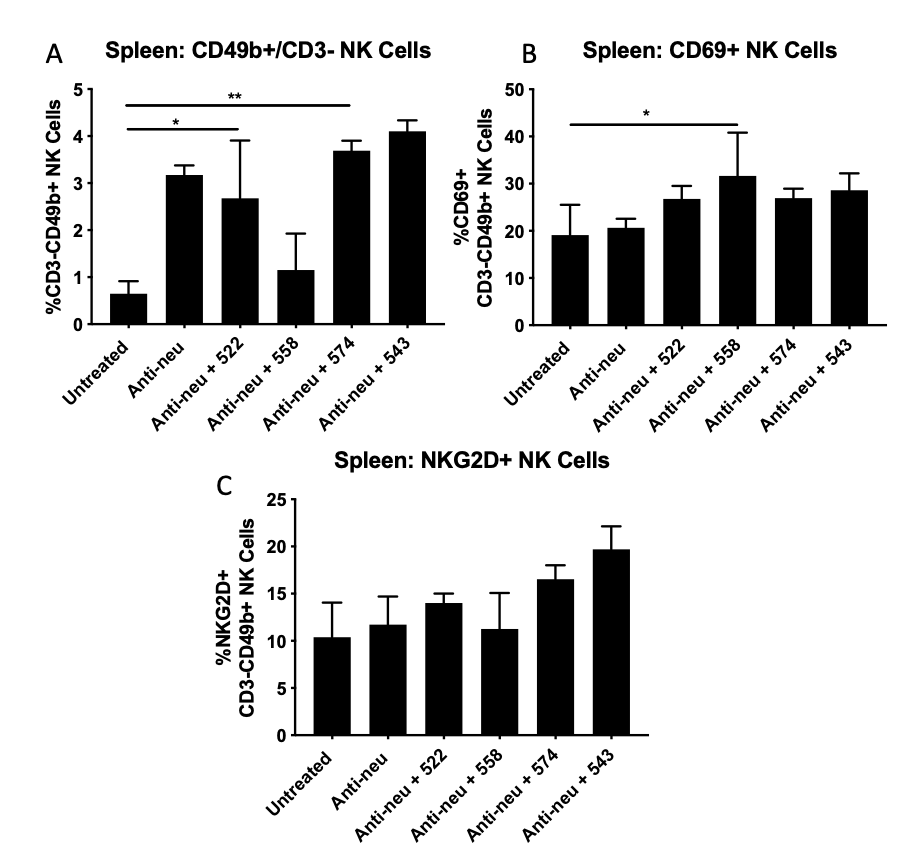
**

**Supplementary Figure 7**: *Ex vivo* analysis of spleen post treatment with anti-HER2/neu antibody and TLR7/8 agonists **(A)** Percentage of CD49b^+^/CD3^-^ NK cells in spleen, *P<0.05, **P<0.01 **(B)** Percentage of CD69^+^ NK cells in spleen, *P<0.05 **(C)** Percentage of NKG2D^+^ NK cells in spleen. All statistical analysis is based on ordinary one-way ANOVA with multiple comparisons.


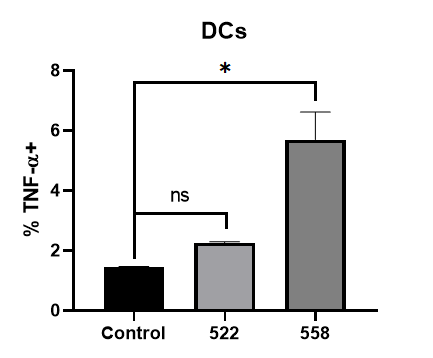

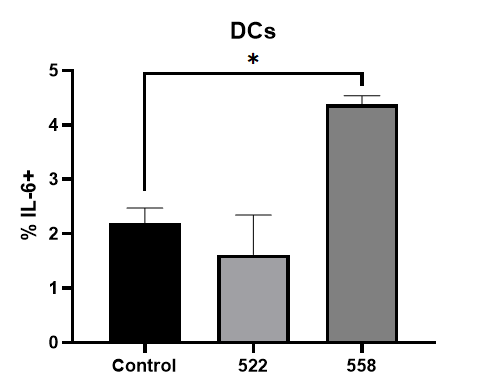

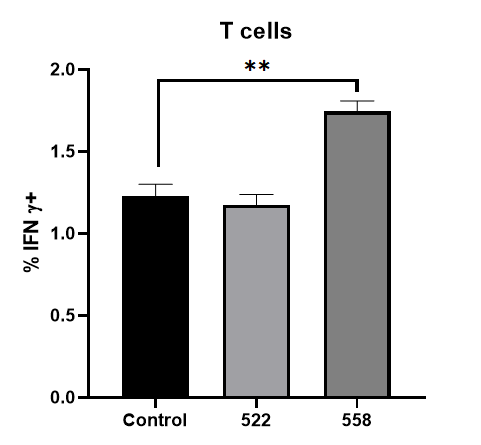


**Supplementary Figure 8**: Human PBMCs were incubated with 522 or 558 (1 μM) for 6 h and intracellular cytokine expression were measured by flow cytometry. Singlets were identified and further plotted on FSC vs SSC axis. A population was selected and gated on the FSC vs CD11c axis (for DCs) or CD3 (for T cells). CD11c/CD3 positive events were analyzed further for cytokine expression. Results are reported as mean ± SD, n=2, *p<0.05 **p<0.01, ns = not significant (p>0.05), One-way ANOVA.
